# A nationwide passive surveillance on fungal infections shows a low burden of azole resistance in molds and yeasts in Tyrol, Austria

**DOI:** 10.1007/s15010-018-1170-0

**Published:** 2018-07-03

**Authors:** Cornelia Lass-Flörl, Astrid Mayr, Maria Aigner, Michaela Lackner, Dorothea Orth-Höller

**Affiliations:** Division of Hygiene and Medical Microbiology, Medical University of Innsbruck, Schöpfstraße 41, 6020 Innsbruck, Austria

**Keywords:** Fungal infections, Resistance, Antifungals, Fungal surveillance

## Abstract

**Purpose:**

To determine the burden of antifungal resistance in fungi over the last 10 years.

**Methods:**

Performance of a semi-nationwide surveillance on antifungal resistance.

**Results:**

We observed a low frequency of azole resistance in *Aspergillus fumigatus*, a moderate increase of echinocandin resistance in yeasts, and a stable amphotericin B activity in yeasts and molds. Posaconazole resistance in *Aspergillus terreus* occurred in a few isolates.

**Conclusion:**

The burden of resistance in fungi seems to be low in Tyrol, Austria.

## Introduction

Invasive fungal infections (IFI) are associated with a high rate of morbidity and mortality in at-risk patients [1]. *Candida* and *Aspergillus* species remain the predominant pathogens with a shift towards resistant fungal pathogens being noted [2]. Reports on resistance prevalence on fungi and bacteria vary, depending on regions, populations, and health-care facilities evaluated [3]. In Austria, a continuous monitoring of antimicrobial resistance is required by law; the idea is to increase the awareness of health-care associated infections, to provide data which support national and international comparison, and at least to provide reference data. Hence, within the last 10 years, we performed a nationwide passive surveillance on antifungal drug resistance and notice a low rate of drug resistance in molds when compared to other countries [4].

## Methods

Clinical samples were obtained from the University Hospital Innsbruck and other medical centres in Tyrol, Austria. Fungal positive, primarily sterile specimens, and body fluids (*n* = 3903) were evaluated with broncho-alveolar lavages (*n* = 1602), blood cultures (*n* = 1580), and tissue biopsies (*n* = 312) being the most important ones.

Fungal specification was determined either by the conventional methods, matrix-assisted laser desorption ionization–time of flight mass spectrometry, or ribosomal internal transcribed spacer DNA sequencing. In total, 2670 yeasts and 1565 molds were detected from routine sampling and were evaluated for their antifungal susceptibility. The Etest^®^ (bioMérieux, Vienna, Austria) and the reference methods released by the European Committee of Antimicrobial Susceptibility Testing for yeasts and molds (EUCAST-AFST) [5, 6] were applied to evaluate the in vitro susceptibility against amphotericin B, anidulafungin, voriconazole, posaconazole, and isavuconazole; MIC results were read following 24 h of incubation, if not, otherwise, indicated. Quality control was performed as recommended in EUCAST documents using *C. krusei* ATCC 6258 and *C. parapsilosis* ATCC 22019. EUCAST-AFST was applied only in cases of imprecise Etest^®^ results and confirmation of resistance. EUCAST antifungal clinical breakpoints (CBPs) and epidemiological cutoffs (ECVs) have been used to monitor the emergence of resistance; these values segregate wild-type isolates from isolates that are likely to carry a resistance mechanism, designated non-wild type. Resistance trends were calculated for species for which in vitro resistance increased > 0.5% within the last 10 years.

## Results

Tables 1, 2 summarize the results of Etest^®^ and/or EUCAST broth microdilution method for echinocandins, azoles, and amphotericin B; Table 3 displays major fungal resistance trends. Amphotericin B was active against a broad range of yeasts and molds tested; resistance is not on rise. Overall, anidulafungin displayed good activity against *Candida* species; resistance increased from 0.5 to 3.6% in *C. glabrata*, from 0.5 to 1.7% in *C. parapsilosis*, and from 0.5 to 0.9% in *C. albicans* isolates tested. Resistance rates of *C. glabrata* against azoles were consistent over the years.

**Table 1:** In vitro susceptibility of the various antifungal agents against molds (*n* = 1233)

Species	No. of isolates	Susceptibility (MIC, µg/ml) of indicated agents to molds									
		AMB		ANI		ISA^a^		VOR		POS	
		MIC50	MIC90	MEC50	MEC90	MIC50	MIC90	MIC50	MIC90	MIC50	MIC90
*Aspergillus* species											
*Aspergillus fumigatus*	338	0.5	2	0.25	1	0.5	1	0.25	1	0.125	0.25
*Aspergillus terreus*	334	2	4	0.125	0.5	0.5	1	0.25	1	0.125	1
*Aspergillus flavus*	121	1	4	0.25	1	0.5	1	0.5	1	0.125	0.5
*Aspergillus niger*	75	0.5	1	0.25	2	2	> 2	0.5	1	0.25	0.5
*A. fumigatus* (azole-resistant)	1	0.5	1	0.25	1	> 2	> 2	> 2	> 2	> 2	> 2
Mucormycetes											
*Rhizomucor* species	77	0.25	2	> 4	> 4	1	> 4	> 4	> 4	1	4
*Lichtheimia corymbifera*	34	1	2	> 4	> 4	1	2	> 4	> 4	0.5	2
*Lichtheimia* species	27	0.5	2	> 4	> 4	0.5	2	> 4	> 4	0.5	1
*Rhizopus microsporus*	13	0.5	2	> 4	> 4	0.5	2	> 4	> 4	0.25	1
*Rhizopus arrizhus*	46	1	2	> 4	> 4	2	4	> 4	> 4	1	2
*Rhizopus* species	22	0.5	2	> 4	> 4	0.5	2	> 4	> 4	1	2
*Mucor hiemalis*	13	0.5	2	> 4	> 4	2	> 4	> 4	> 4	1	2
*Mucor* species	19	0.5	2	> 4	> 4	1	2	> 4	> 4	0.5	4
*Cunninghamella* species	24	0.5	4	> 4	> 4	0.5	2	> 4	> 4	0.5	2
Others											
*Scedosporium prolificans*	13	> 4	> 4	4	> 4	> 4	> 4	> 4	> 4	> 4	> 4
*Scedosporium apiospermum*	9	2	> 4	1	> 4	2	> 4	2	> 4	0.5	4
*Penicillium* species	32	0.5	1	> 4	> 4	0.5	> 4	2	> 4	0.5	2
*Fusarium solani*	23	4	4	> 4	> 4	4	> 4	> 4	> 4	1	1
*Fusarium oxysporum*	12	1	4	> 4	> 4	1	4	2	> 4	0.5	2

Minimal inhibitory concentration (MIC) and minimal effective concentration (MEC) are given; MIC 50 and MIC 90 display the MICs inhibiting 50 and 90% of isolates

^a^ *AMB* amphotericin B, *ANI* anidulafungin, *ISA* isavuconazole, *VOR* voriconazole, *POS* posaconazole

ISA was tested in only few isolates, as was introduced in routine in 2016

**Table 2:** In vitro susceptibility of the various antifungal agents against yeasts species (*n* = 2670)

Species	No. of isolates	Susceptibility (MIC, µg/ml) of indicated agents to yeasts									
		AMB		ANI		ISA^a^		VOR		POS	
		MIC50	MIC90	MIC50	MIC90	MIC50	MIC90	MIC50	MIC90	MIC50	MIC90
*Candida* species											
*Candida albicans*	1259	0.25	1	0.06	0.125	0.008	0.015	0.006	0.125	0.064	0.25
*Candida glabrata*	718	0.5	1	0.06	0.5	0.5	4	0.25	4	0.5	4
*Candida parapsilosis*	320	0.5	1	0.5	4	0.125	2	0.03	1	0.06	2
*Candida krusei*	259	0.5	2	0.03	0.5	0.5	4	0.25	2	0.5	2
*Candida lusitaniae*	29	0.5	1	0.5	1	0.03	0.25	0.006	0.5	0.06	0.125
*Candida tropicalis*	21	0.25	1	0.03	0.5	0.06	1	0.03	1	0.06	0.25
*Candida guilliermondii*	17	0.5	1	1	1	0.25	1	0.06	0.5	0.125	0.25
Others											
*Saccharomyces cerevisiae*	21	0.5	1	2	2	0.5	4	0.125	2	0.25	4
*Cryptococcus neoformans*	10	1	1	> 4	> 4	0.06	0.125	0.06	0.125	0.06	0.5
* Trichosporon species	9	0.5	1	> 4	> 4	0.125	1	0.06	0.5	0.25	1
*Geotrichum candidum*	7	1	2	2	4	0.5	1	0.125	0.5	0.25	0.5

*Trichosporon species includes *T. asahii* and *T. ovoides*

Minimal inhibitory concentration (MIC) are given; MIC 50 and MIC 90 display the MICs inhibiting 50 and 90% of isolates

*AMB* amphotericin B, *ANI* anidulafungin, *ISA* isavuconazole, *VOR* voriconazole, POS posaconazole

^a^ISA was tested in only few isolates, as was introduced in routine in 2016

**Table 3:** Resistance trends (species for which in vitro resistance increased for > 0.5% within the last 10 years) applying EUCAST breakpoints

Species	Drug	% resistance			
		2007–2009	2010–2012	2013–2015	2016–2017
*Aspergillus terreus*	Posaconazole	0.3	0.6	0	0
*Candida albicans*	Voriconazole and/or posaconazole	0.5	0.8	0.6	0.9
*Candida glabrata*	Anidulafungin^a^	0.5	1.7	2.9	3.6
*Candida parapsilosis*	Anidulafungin^a^	0.5	0.8	1.1	1.7

^a^Indicator drug to be tested according to EUCAST

The absence of ECVs and CBPs for rare species such as *Candida lusitaniae* and *C. guilliermondii* does not support a categorization in wild types and non-wild types; however, an increase of isolates with high MICs over time was not recorded.

ECVs were also recently published from EUCAST for various *Aspergillus* species, and we applied these criteria for *A. fumigatus* species complex, *Aspergillus niger* species complex, *Aspergillus terreus* species complex, and for *Aspergillus flavus* species complex isolates. Only one azole-resistant *A. fumigatus* isolate was detected; azole resistance in *A. terreus* was noticed in a few isolates, which were collected between 2007 and 2009. ECVs and CBPs are lacking for non-*Aspergillus* molds, and hence, we display MIC data only, see Table 1.

## Discussion

This survey on antifungal resistance observed a low frequency of azole resistance in *A. fumigatus*, a moderate increase of echinocandin resistance in yeasts, and a stable amphotericin B activity against yeasts and molds. Posaconazole resistance in *A. terreus* was newly detected in a few isolates; however, these isolates were collected between 2007 and 2009. Some countries report an alarmingly increase of antifungal resistance development within the past, with azole-resistant *A. fumigatus* displaying the major threat [4]. In Tyrol, fungal in vitro susceptibility data have not changed tremendously; conspicuous trends were not observed. Susceptibility tests were performed by applying the Etest^®^ method to determine the MICs of the respective antifungal agents; in uncertain cases, the EUCAST methodology was applied [5, 6]. This commercially available test was previously described as a simple and reliable method for AST that was comparable in performance to reference procedures [7].

Most interesting from our study is the fact that we did not observe azole resistance in *A. fumigatus* as a major issue; only a few isolates were identified within the last 10 years. This is contrary to the overall worldwide trend. Azole antifungal drugs are the first line of therapy against *A. fumigatus*, a common etiologic agent of aspergillosis [8]. Resistance to azole drugs has been associated with treatment failure and deaths in patients with aspergillosis [4]. However, azole resistance in *A. fumigatus* has been documented in many regions in the past decade. Why neighbouring countries and others display such increase of azole-resistant *A. fumigatus* is unknown yet; it is hypothesized that the environment displays major sources due to agricultural usage of azoles [4].

Applying the new EUCAST breakpoints categorized a few isolates of *A. terreus* species complex to be posaconazole resistant with the clinical importance being unclear; a CBP of > 0.25 µg/ml for posaconazole was applied, and most of the patients involved were treated with voriconazole and improved [2]. At our institution, infections due to *A. terreus* were prevalent over the last 3 decades; however, the timely use of targeted treatment with voriconazole and the widespread use of posaconazole/micafungin prophylaxis decreased invasive aspergillosis from 12 to 6% in patients with haematological malignancies [9]; fungal breakthrough infections due to *A. terreus* are lacking [2], and hence, the role of resistance against posaconazole in *A. terreus* needs further clinical and molecular-based evaluation.

*C. albicans* is the leading cause of candidemia worldwide being responsible for one-third of the cases of candidemia in the US and European countries [10]. The echinocandin resistance rates among the *Candida* species were low overall, but resistance among *C. glabrata* is on rise [10]. Such development supports the importance of antifungal stewardship programs, as the echinocandins are the drug of choice to treat *Candida*-infections.

Remarkable is the efficacy of amphotericin B against this broad panel of fungi without either losing activity or introducing resistance over time. Amphotericin B MIC data are stable, resistance development is lacking. Hence, this drug has an exceptional position within the antifungals available in terms of resistance development.

For the mucormycetes and other molds, ECVs and CBPs are lacking; whether MICs > 2 mg/ml for amphotericin B, posaconazole, and isavuconazole represent clinical failure is unclear and needs further investigation. However, the proportion of fungi displaying MICs > 2 mg/ml for amphotericin B and posaconazole was stable over the last decade. By contrast, higher isavuconazole MICs against the Mucorales were not uncommon, see Table 1. This finding is of great interest, as isavuconazole was recently licensed as the second-line treatment of mucormycoses on the European market. Hence, more real life data are needed to clinically define azole resistance in Mucorales.

Limitations of this study include that data acquisition over years is a subject to change. Hence, different experts may read test results a little differently; protocols and the arbitrary nature of sampling may have changed minimally.

Despite the low antifungal resistance rates among *Candida* and *Aspergillus* isolates, continuous monitoring of antifungal susceptibility patterns and continuous determination of an understanding of mechanisms of resistance seem prudent. Reports of breakthrough infections, the increasing prevalence of uncommon species refractory to clinically available antifungal agents, and emerging resistance mechanisms highlight the importance of local and global surveillance.
